# Targeting Accuracy, Procedure Times and User Experience of 240 Experimental MRI Biopsies Guided by a Clinical Add-On Navigation System

**DOI:** 10.1371/journal.pone.0134370

**Published:** 2015-07-29

**Authors:** Harald Busse, Tim Riedel, Nikita Garnov, Gregor Thörmer, Thomas Kahn, Michael Moche

**Affiliations:** Department of Diagnostic and Interventional Radiology, Leipzig University Hospital, Leipzig, Germany; Shenzhen institutes of advanced technology, CHINA

## Abstract

**Objectives:**

MRI is of great clinical utility for the guidance of special diagnostic and therapeutic interventions. The majority of such procedures are performed iteratively ("in-and-out") in standard, closed-bore MRI systems with control imaging inside the bore and needle adjustments outside the bore. The fundamental limitations of such an approach have led to the development of various assistance techniques, from simple guidance tools to advanced navigation systems. The purpose of this work was to thoroughly assess the targeting accuracy, workflow and usability of a clinical add-on navigation solution on 240 simulated biopsies by different medical operators.

**Methods:**

Navigation relied on a virtual 3D MRI scene with real-time overlay of the optically tracked biopsy needle. Smart reference markers on a freely adjustable arm ensured proper registration. Twenty-four operators – attending (AR) and resident radiologists (RR) as well as medical students (MS) – performed well-controlled biopsies of 10 embedded model targets (mean diameter: 8.5 mm, insertion depths: 17-76 mm). Targeting accuracy, procedure times and 13 Likert scores on system performance were determined (strong agreement: 5.0).

**Results:**

Differences in diagnostic success rates (AR: 93%, RR: 88%, MS: 81%) were not significant. In contrast, between-group differences in biopsy times (AR: 4:15, RR: 4:40, MS: 5:06 min:sec) differed significantly (p<0.01). Mean overall rating was 4.2. The average operator would use the system again (4.8) and stated that the outcome justifies the extra effort (4.4). Lowest agreement was reported for the robustness against external perturbations (2.8).

**Conclusions:**

The described combination of optical tracking technology with an automatic MRI registration appears to be sufficiently accurate for instrument guidance in a standard (closed-bore) MRI environment. High targeting accuracy and usability was demonstrated on a relatively large number of procedures and operators. Between groups with different expertise there were significant differences in experimental procedure times but not in the number of successful biopsies.

## Introduction

Minimally invasive diagnostic and therapeutic procedures are typically performed under image guidance. Established imaging techniques like ultrasound (US) and computed tomography (CT) are widely available and allow for fast or even real-time control of the procedure. In special cases, however, MRI becomes the method of choice, most often when targets or critical anatomical structures along the access path are only visible by MRI [1]. The last two decades have seen a number of dedicated MRI systems, in particular open units that provide good access to the patient and have been successfully used for image-guided procedures [2–5]. The ongoing development of faster pulse sequences has largely contributed to make instrument navigation more intuitive [6,7]. At the same time, traditional 60-cm bore MRI systems have increasingly been replaced by wide-bore models that offer some more space for operation [8–12].

Some interventions, often in well-defined regions of the body, can actually be performed inside the magnet, whereas the most common practice with cylindrical MRI systems is to slide the patient in and out of the magnet for successive imaging and intervention steps, respectively [11,13,14]. This gives the operator unobstructed access to the patient but is generally time-consuming and prone to positioning errors. Guidance can be improved by providing the operator with an overlay of the instrument position on continuously reformatted MRI data. In one implementation [15], this was achieved by a navigation system with optical real-time instrument tracking. The system is characterized by a floating reference structure that enables navigation for procedures in practically all parts of the body. It has already been applied clinically for various percutaneous interventions, mainly biopsies, in different target regions, for example, the shoulder, breast, liver, paravertebral region, pelvis or the femur. Corresponding information for some of these cases can be found in the literature [8,15,16]. In comparison with other techniques, continuous registration is ensured by a fast, one-time 3D localization of three smart MR markers that can be reliably detected over a wide variety of imaging conditions. The setup is invariant against both motion of the patient table as well as that of the rolling 3D digitizer. Any multislice or 3D data can be loaded for planning purposes and control images can be automatically prescribed along any optically measured instrument pose [15].

Detailed evaluation of the usability and workflow of a new enabling technology is generally difficult. Clinical studies obviously provide more meaningful parameters but are often limited by small case numbers, an uncontrolled procedural complexity and results from properly trained physicians only. Experimental results, on the other hand, need to be interpreted more carefully but also provide the unique opportunity to improve statistical power, minimize unwanted variation by standardized operating conditions, and deliberately determine the impact of user expertise, in our case, on targeting accuracy and procedure times.

The goal of this work was therefore a comprehensive experimental assessment of the overall targeting accuracy, usability and workflow of such a navigational tool by analyzing a large number of phantom biopsies performed by medical operators with different levels of expertise.

## Materials and Methods

### Hardware Components and Setup

**Fig 1:**
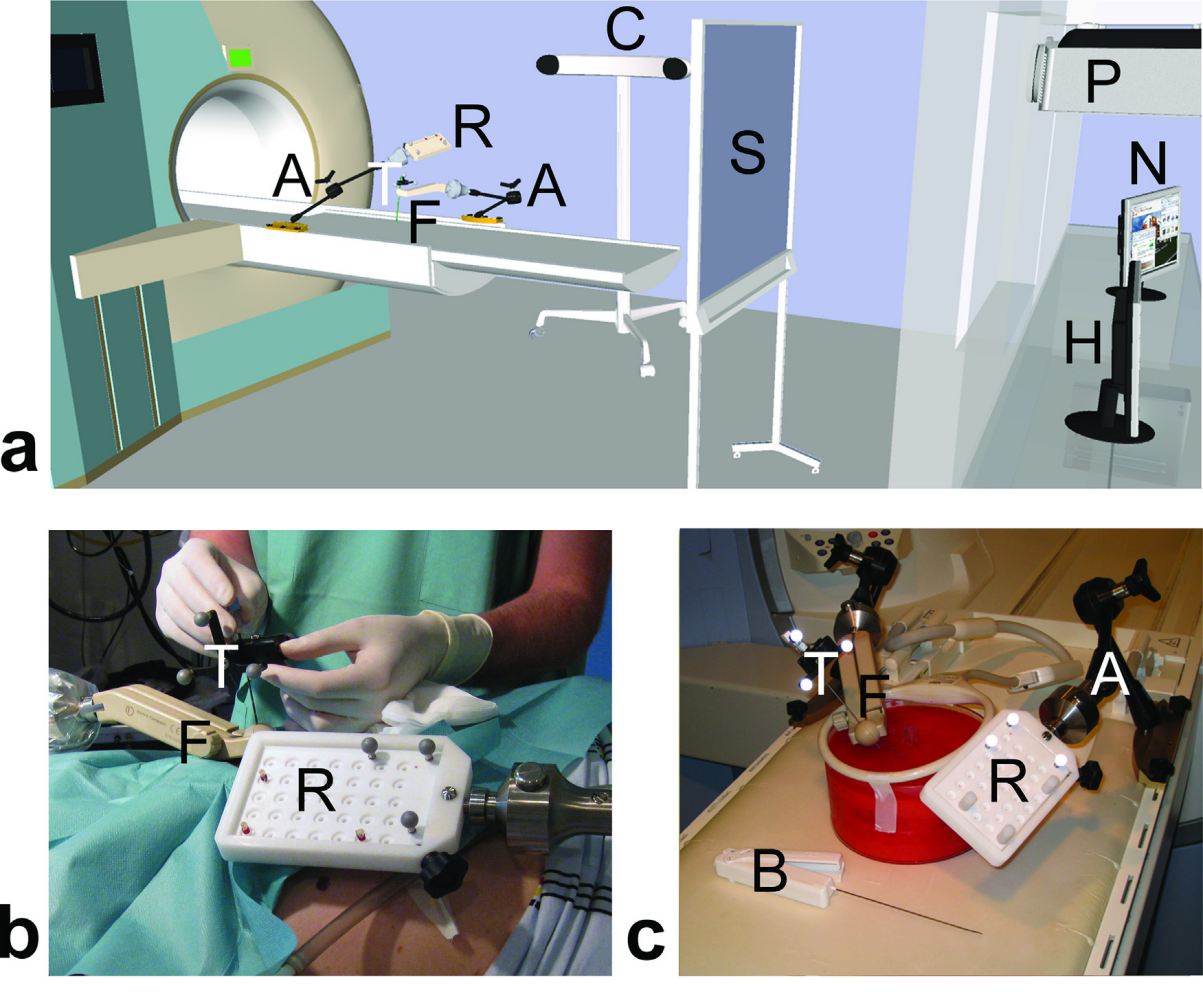
Add-on navigation solution for MRI-guided interventions. (a) Schematic drawing of the overall setup illustrating the components at the MRI table ("A" articulating arm, " R" reference board, "F" front-end module for alignment; "T" tracker for instrument), in the MR room ("C" optical 3D tracking camera, "S" projection screen) and in the control room ("P" LCD projector, "N" navigation workstation, "H" MR host computer). (b) Clinical setup of an MRI-navigated biopsy in the kidney. (c) Experimental setup used for the assessment of targeting accuracy, procedure times and usability ("B" biopsy gun).

Fig 1 gives an overview of the hardware components and setup of the navigation system for MRI-guided interventions. In short, the position, orientation, and motion of a medical instrument, e. g., biopsy gun or coaxial needle, can be followed in a multiplanar virtual MRI scene that is displayed on a nearby screen. A compact reference device with a set of three reflective markers needs to be attached to the instrument. An optical 3D digitizer (Polaris Spectra, NDI, Waterloo, ON, Canada) then tracks this instrument relative to a second reference set on a board that remains fixed to the patient table. This board also features a set of MR markers [17] that are used for registration of the navigation scene. A special guiding device (front-end module) is used to hold, adjust and lock the coaxial needle in the intended biopsy position. Further details about the navigation principle can be found in a previous work [15].

### Biopsy Phantom

**Fig 2:**
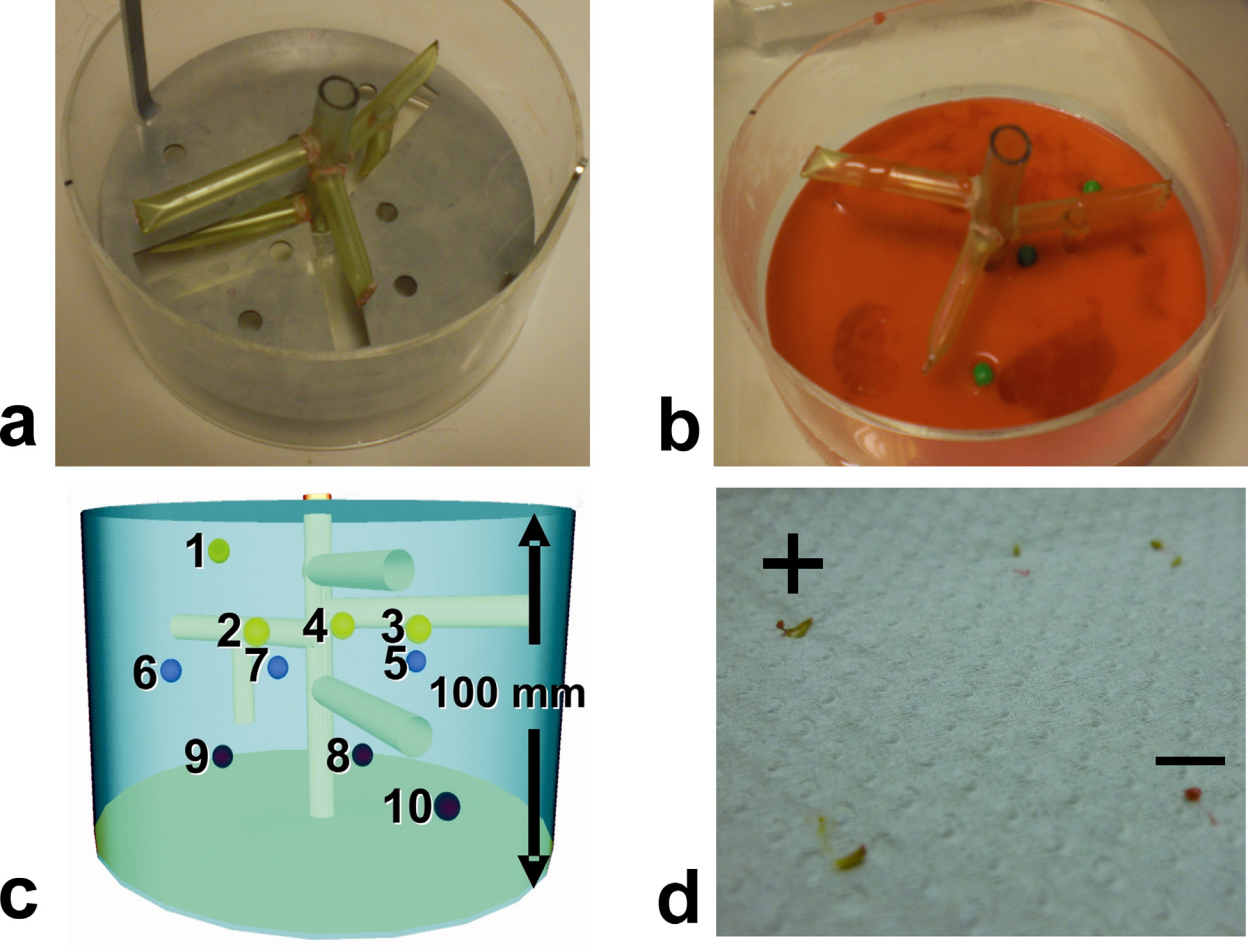
Standardized biopsy phantom. (a) Acrylic glass cylinder (10 cm high and 20 cm in diameter) with model vasculature and target positioning template. A three-dimensional structure of silicone tubes served as model vasculature that needed to be avoided. Custom-drilled holes inside a stainless steel disc ensured that experimental targets were placed in the same 3D positions for all trials. (b) The tissue phantom was made out of ordinary red cake glaze in double concentration (160 g powder per 400 ml of water). Ten ordinary green peas were embedded as biopsy targets. The surface of the hardened glaze was later impregnated with a virucidal hand disinfectant (Sterilium Viruguard) and the phantom was stored in a refrigerator. (c) Target difficulty varied with insertion depth (four distinct levels between 17–76 mm from the phantom surface) and placement with respect to the vasculature. Three targets (# 4, 7 and 9) were located under a vessel or another target and could only be accessed by an oblique path. (d) Photograph of tissue samples taken with the fully automatic biopsy gun. Biopsy was counted as success when sample contained green material from the pea (+ sign). Samples of base material (cake glaze) appeared red (–sign).

A standardized biopsy phantom was used for all trials (Fig 2). It consisted of plain, opaque glaze inside a large acrylic glass cylinder (10 cm high and 20 cm in diameter). Ten soft green peas were used as biopsy targets. The average diameter was determined with a caliper on 100 randomly selected peas. A geometric template disc was used to reproducibly arrange the peas in 3D for all trials. Artificial vascular structures were embedded in the phantom so that the interventionalist would have to navigate around these structures while performing the biopsies. The level of targeting complexity varied with the insertion depth of the targets and with the potential presence of vessel structures along the shortest access path perpendicular to the phantom surface (Fig 2).

### Operators

Biopsies were performed by 24 operators with different interventional expertise: 8 attending (AR) and 8 resident radiologists (RR) from the Diagnostic and Interventional Radiology Department as well as 8 medical students (MS) from the local School of Medicine. A 6-minute instruction video was presented to each operator immediately before the biopsies. One resident radiologist had clinical experience with this navigation system; all other operators had never used it before. Our clinical study evaluating the safety and workflow of the used system was approved by the IRB committee of the Leipzig University Faculty of Medicine. The present phantom study does not involve any healthcare interventions on a person. All operators participated voluntarily in this study and were informed that they could opt out without penalty.

### MR Imaging

The biopsies were performed with a 60-cm closed-bore 1.5-T MRI system (Magnetom Symphony, Siemens Healthcare, Erlangen, Germany). Prior to the biopsies, the phantom setup was registered to MRI coordinates by a one-time acquisition of MR-marker images and a subsequent, fully automatic 3D localization (15–17) of the marker signals. A balanced steady-state free precession (SSFP) sequence with large volume coverage in three orthogonal views (axial, coronal, sagittal) was used for marker imaging (TR/TE = 6.8/2.8 ms, single slice with thickness = 300 mm, field of view FOV = 300 mm × 300 mm, acquisition matrix = 512 × 512, flip angle = 0.8°, receiver bandwidth = 222 Hz/pixel, total acquisition time = 10.5 s, and manufacturer's distortion correction enabled). Data from a T1-weighted volume-interpolated breath-hold examination (VIBE) were used for anatomic imaging (TR/TE = 3.8/1.7 ms, 60 slices with thickness = 2.0 mm, FOV = 210 mm × 210 mm, acquisition matrix = 256 × 167, flip angle = 15°, total acquisition time = 23 s).

### Navigation PC

**Fig 3:**
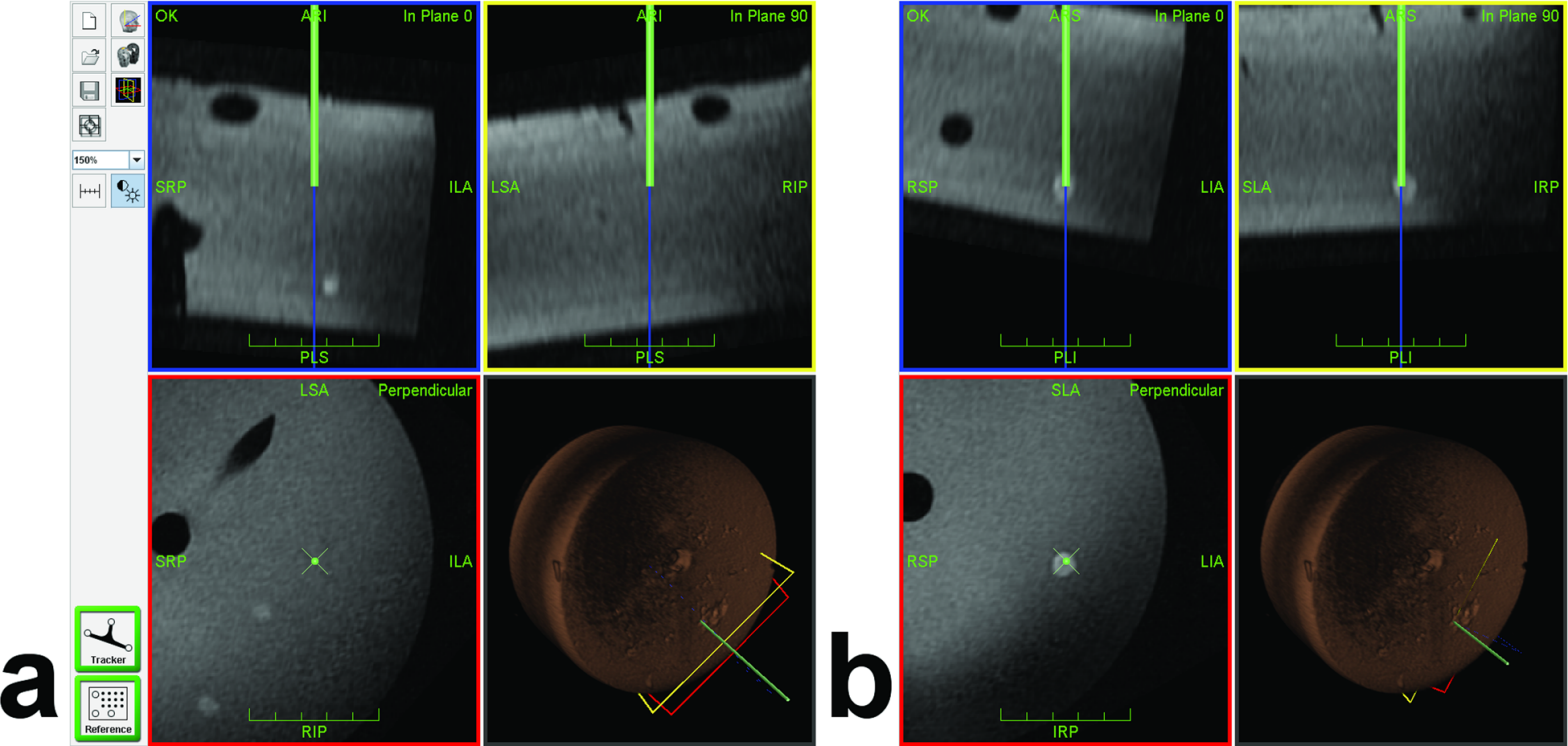
Navigation Interface During Target Approach. The navigation interface continuously displays three orthogonal planes reconstructed from the planning MRI dataset along (in-plane 0° and in-plane 90°, blue and yellow frames) and perpendicular (red frame) to the tracked needle. The virtual needle and its extension are displayed as green and blue lines, respectively (virtual tip at the end of the green line). The fourth view indicates the 3D position of the needle and all viewing planes with respect to the volume-rendered MRI dataset. Phantom vessels (tubes) and targets (peas) appear as hypointense and hyperintense structures, respectively. (a) Sample screenshot of a partially inserted needle with the virtual extension slightly off target (#10, in plane 0°). Two other targets (#6 and #7) happen to appear in the perpendicular view. (b) Later screenshot after corresponding adjustment showing the virtual needle tip inside the target (in all views).

MRI data from the MR console were manually sent to the navigation PC. Automatic marker localization [18] was handled by a stand-alone application written under IDL (Exelis Visual Information Solutions, Boulder, CO, USA). The navigation software [15] featured modules for graphical access planning and navigation (Fig 3). Phantom registration was only needed once per session and simply involved the measured MR-marker coordinates as well as the fixed 3D geometries of tracker and markers to automatically compute the transformation matrix between tracked instrument and MR coordinates.

### Biopsy Cycle

Each operator performed 10 successive biopsy cycles in the same order (Fig 2). A biopsy cycle consisted of five individual steps. During access planning (i), the operator moved the coaxial needle (16G, 135 mm long, Invivo Germany, Schwerin) with the attached tracker over the phantom and followed the corresponding navigation scene on the in-room projection screen to identify the next target and define a corresponding access path. Arm adjustment (ii) involved the rough positioning along that access path and locking of the articulating arm above the phantom. The navigation step (iii) comprised insertion of the coaxial needle into the front-end module, alignment of the virtual needle tip with the center of the MR-visible pea on the navigation screen (Fig 3) and accurate fixation of the instrument. In the sampling step (iv), the biopsy gun (18G, 175 mm long, Invivo Germany) was cocked, inserted into the coaxial needle and released for sample collection. For control imaging (v), the biopsy instrument remained in place. The MR table was moved into the scanner, the phantom was imaged with the control sequence (see above) and the table was moved back out for the next biopsy cycle. A biopsy was regarded as diagnostic success if the sample clearly contained green material from the pea (Fig 2). For each simulated biopsy, we recorded the partial times for (i) access planning, (ii) arm adjustment, (iii) navigation, (iv) sampling, and (v) control. Each operator was asked to rate 13 items (Table 1) related to the usability and workflow of the system on a Likert scale from strong (5) and basic (4) agreement via indifference (3) to basic (2) and strong (1) disagreement. These questions are summarized in Table 1 along with the results.

**Table 1:** Mean Likert item scores on usability and workflow of navigation tool by 24 operators from three groups.

Question	All	Rank	AR *	RR *	MS *
Q01—I have understood how the navigation system works.	4.89	1	4.75	4.88	4.75
Q12—I would use the system again.	4.75	2	4.75	4.88	4.63
Q09—The system provides additional safety.	4.67	3	4.63	4.88	4.50
Q05—I had the impression that biopsies became easier with the number of targets.	4.67	4	4.50	4.75	4.75
Q11—Biopsies would *not* be simpler without the navigation scene on the screen. ^†^	4.67	4	4.75	4.50	4.75
Q08—The time required to perform a biopsy with that system is *not* too long. ^†^	4.58	6	4.50	4.63	4.63
Q10—The extra technical efforts needed are justified in view of the benefit.	4.42	7	4.50	4.38	4.38
Q13—I have confidence in this technique.	4.21	8	4.25	4.13	4.25
Q02—I can easily orient myself and guide the needle by looking at the navigation screen.	4.13	9	4.13	4.38	3.88
Q03—It is easy to mentally transfer the images on the navigation screen to the real world.	3.92	10	3.88	4.38	3.50
Q04—Operation of the navigation system is self-explaining.	3.88	11	3.88	3.75	4.00
Q06—The articulating module enables easy adjustment and fixation of the biopsy needle.	3.67	12	3.13	4.00	3.88
Q07—The navigation system is *not* susceptible to external perturbations. ^†^	2.79	13	2.88	3.25	2.25
Mean over all items	4.24		4.19	4.37	4.16

* AR: attending radiologists, RR: resident radiologists, and MS: medical students

^†^ opposite item with reverse score, original item was negatively keyed

### Statistical Analysis

Sampling success rates were analyzed with a one-way ANOVA with factor group. Partial times for individual working steps as well as total biopsy-cycle times were analyzed with a one-way, repeated measures ANOVA with factor (operator) group (RR, AR, MS) using the average time of all group members for a given target. Total biopsy times were also analyzed with a one-way, repeated measures ANOVA across all operators with factor target (#1 - #10). User ratings were analyzed with the same test using the score average for all group members for a given item. Negatively-keyed items were scored reversely. If the main effect was significant, post-hoc Bonferroni results were considered. The assumption of sphericity was validated with Mauchly’s test. All tests were performed with SPSS Version 20.0 (IBM Corp., Armonk, NY).

## Results

### Biopsy Phantom

The viscoelastic properties of the phantom material were subjectively considered to be similar to those of biological tissues. The red glaze alone was so opaque that peas were invisible for the operator. They had an average diameter of 8.5 ± 0.5 mm (mean ± SD) with individual values in the 7.0–9.5 mm range. The green pea material collected with the biopsy gun was clearly discernible from the red base material (Fig 2). A single phantom was used by up to six operators because it could be preserved for several weeks. A new phantom was built whenever previous needle insertions left clear traces or voids in the material or when the entire phantom started to decay.

### Success Rates and Biopsy Times for Different Groups

**Fig 4:**
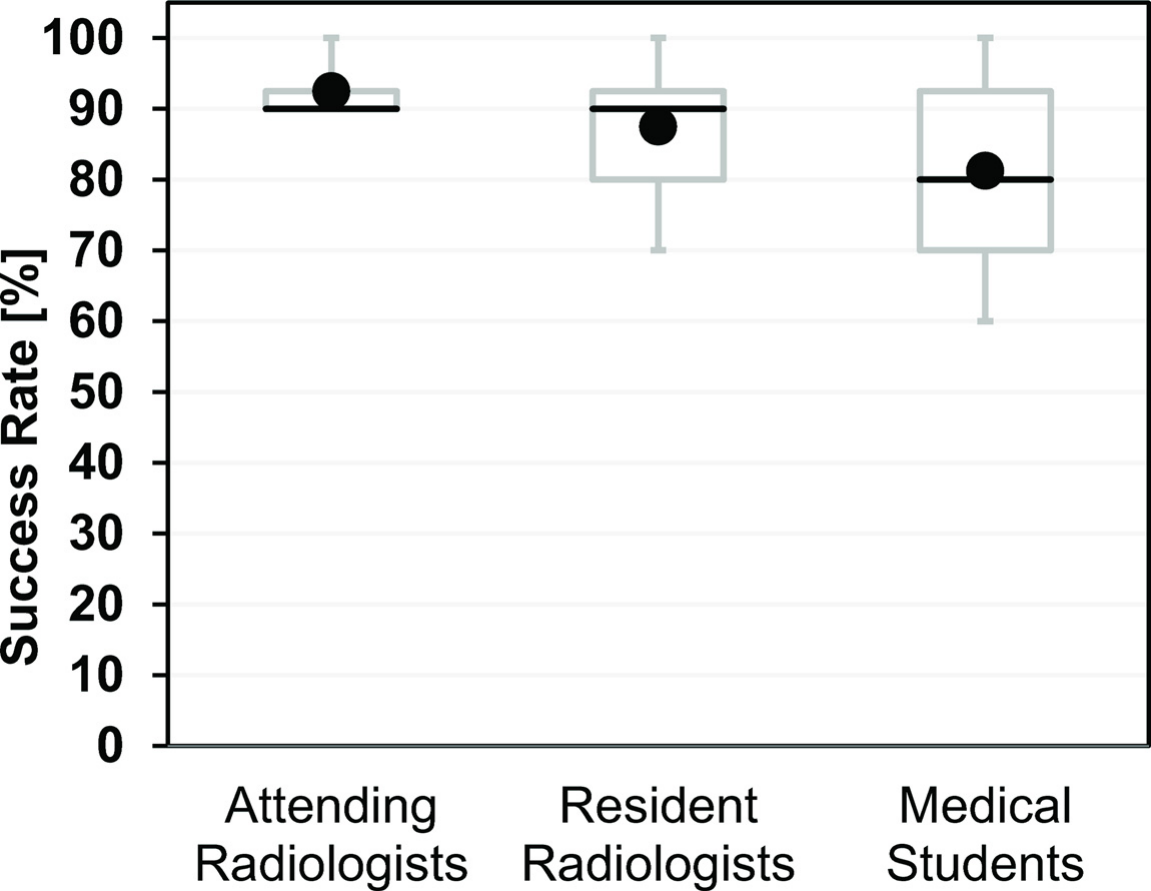
Success rates of experimental biopsies performed by 24 operators from three different groups (AR, RR, MS). Each operator performed 10 trials and the individual success rate was simply the number of successful biopsies multiplied by 10%. Each box with whiskers represents the summary for 8 operators in each group. The gray box stretches from the first (25%, Q1) to the third (75%, Q3) quartile and the black line indicates the second (50%, Q2) quartile (median). Owing to the low number of operators per group (n = 8), the whiskers correspond to the minimum and maximum success rates observed for a single operator. The black circles indicate the mean success rates. Please note that minimum, Q1, and Q2 of the success rate coincide at 90% for the group of attending radiologists. Between-group differences in the mean success rate were statistically not significant (p = 0.132).

**Fig 5:**
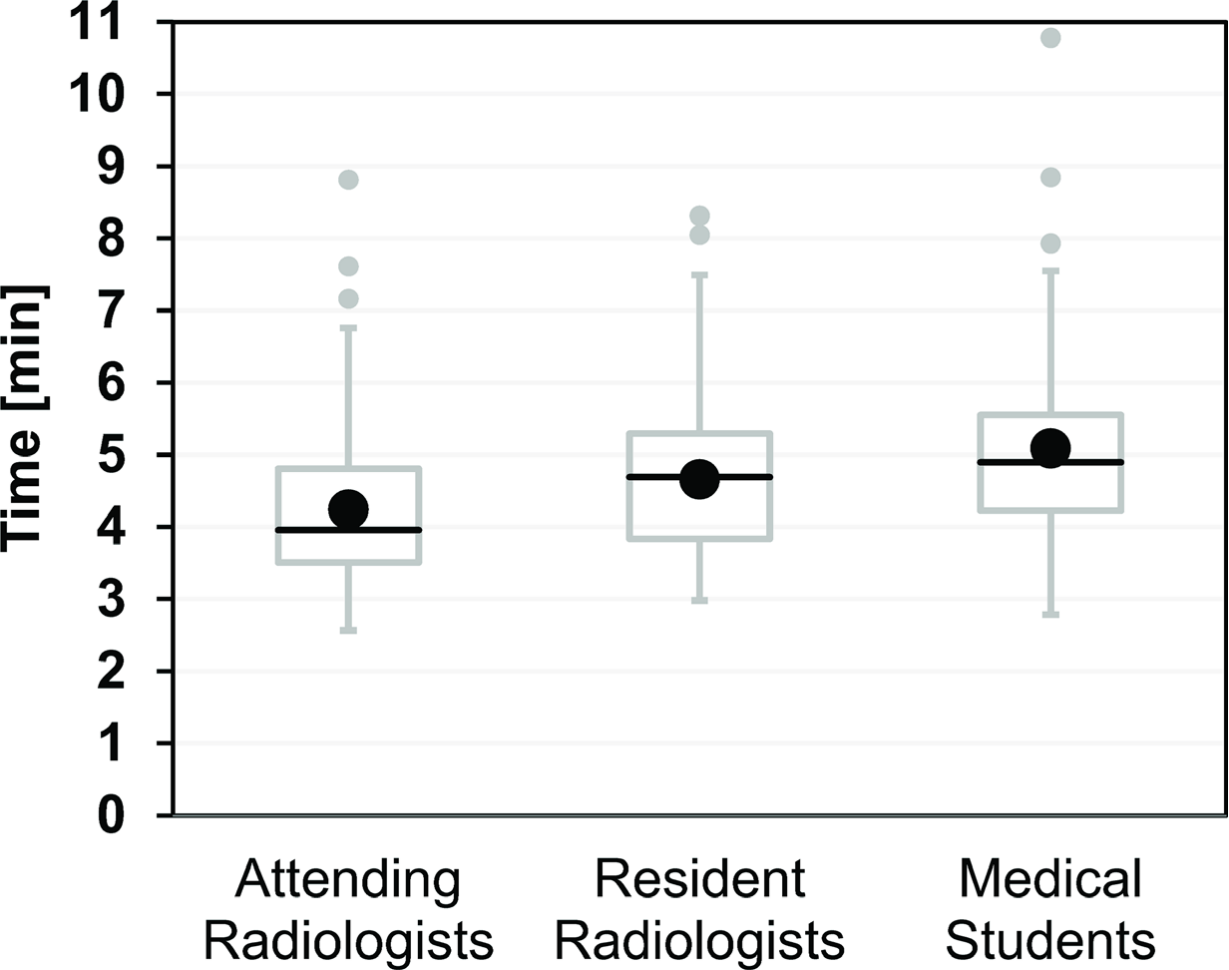
Experimental total biopsy times for 24 operators from three different groups (AR, RR, MS). Each operator performed 10 biopsy cycles in the same order. Each box with whiskers represents the summary of 80 biopsy times from 8 operators. The gray box and black line indicate Q1–Q3. Whiskers were plotted according to the definition by J. W. Tukey and either indicate the last values a maximum of 1.5 × IQR (interquartile range = Q3–Q1) away from Q1 and Q3, respectively, or the minimum and maximum, whichever comes first. Each group had 2–3 upper outliers (gray circles) with times greater than Q3 + 1.5 × IQR. The black circles indicate the mean biopsy times, which were significantly different between groups (p < 0.01). Post-hoc Bonferroni testing revealed significant differences between AR and RR as well as AR and MS.

**Fig 6:**
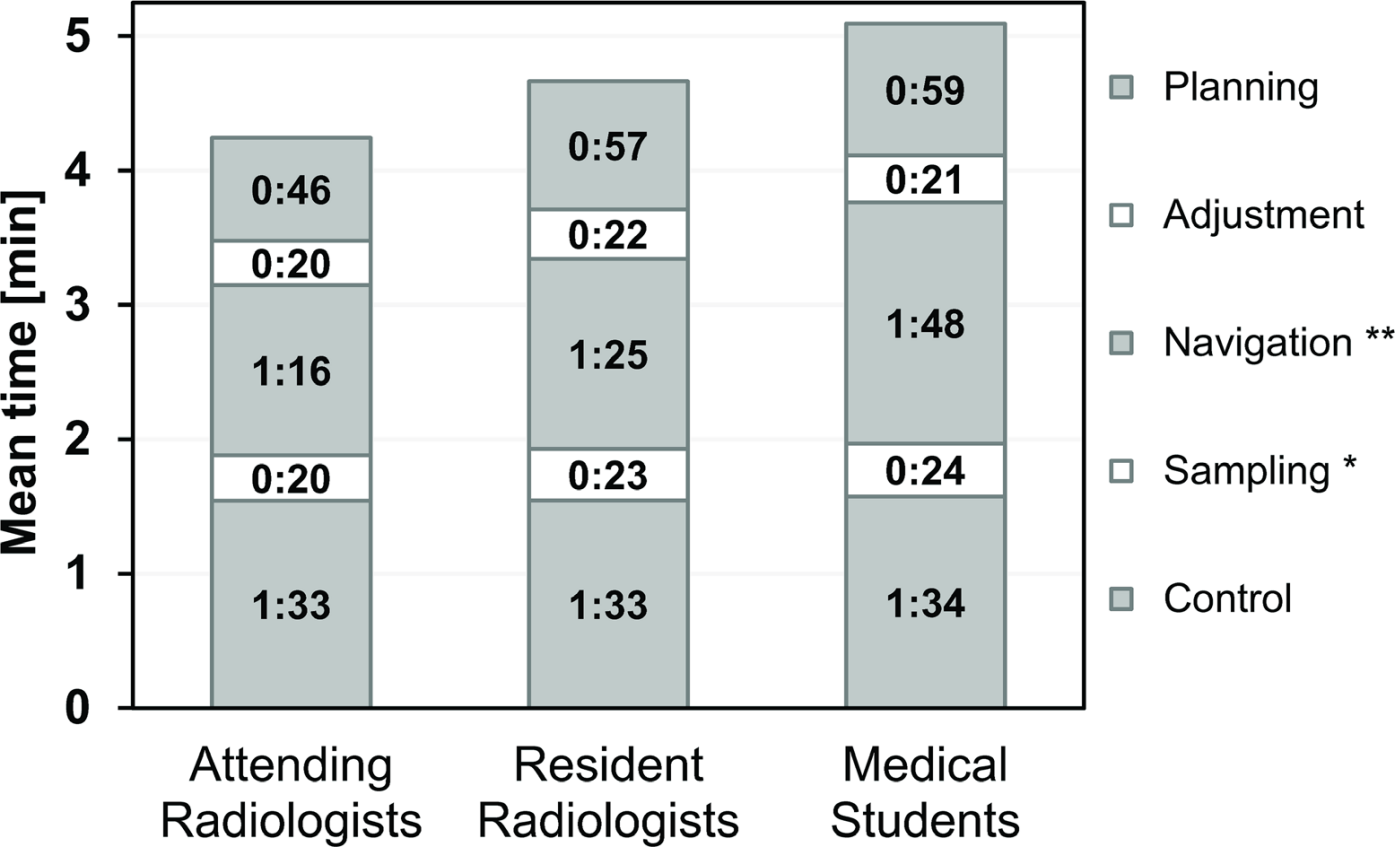
Contribution of five working steps (planning, adjustment, navigation, sampling and control) to the mean experimental biopsy time for each operator group (AR, RR, MS). The times stated are averages of 80 individual measurements from 8 operators on 10 targets each. Navigation times ranged between 01:16 (AR) and 01:48 (MS) and differences between operator groups were significant (ANOVA p < 0.01, **). Sampling times ranged between 00:20 (AR) and 00:24 (MS) and between-group differences were significant (p < 0.05, *). All other working step times were not significantly different (see text for details).

The distribution of the number of hits was 6×9 and 2×10 for attending radiologists (AR), 1×7, 2×8, 3×9 and 2×10 for resident radiologists (RR), and 1×6, 2×7, 2×8, 1×9 and 2×10 for medical students. This corresponds to mean success rates (95% confidence intervals) of 92.5% (88.6–96.4%) for AR, 87.5% (78.8–96.2%) for RR, 81.3% (69.1–93.4%) for MS (Fig 4), and 87.1% (82.3–91.8%) for all 24 operators with no significant differences between them (p = 0.132). In contrast, the mean times for a complete biopsy cycle in minutes and seconds (AR: 04:15, RR: 04:40, MS: 05:06) were significantly (p < 0.01) different (Fig 5). The breakdown of the total times into individual steps for each operator group is shown in Fig 6. The largest absolute time differences between groups were observed for the navigation step (AR: 01:16, RR: 01:25, MS: 01:48, p < 0.01). Differences in the mean sampling times were also significant (p < 0.05) with a maximum of about 4 s between AR and MS. All other time differences were insignificant (planning: p = 0.289, adjustment: p = 0.512, control: p = 0.327).

### Biopsy Times for Different Targets

**Fig 7:**
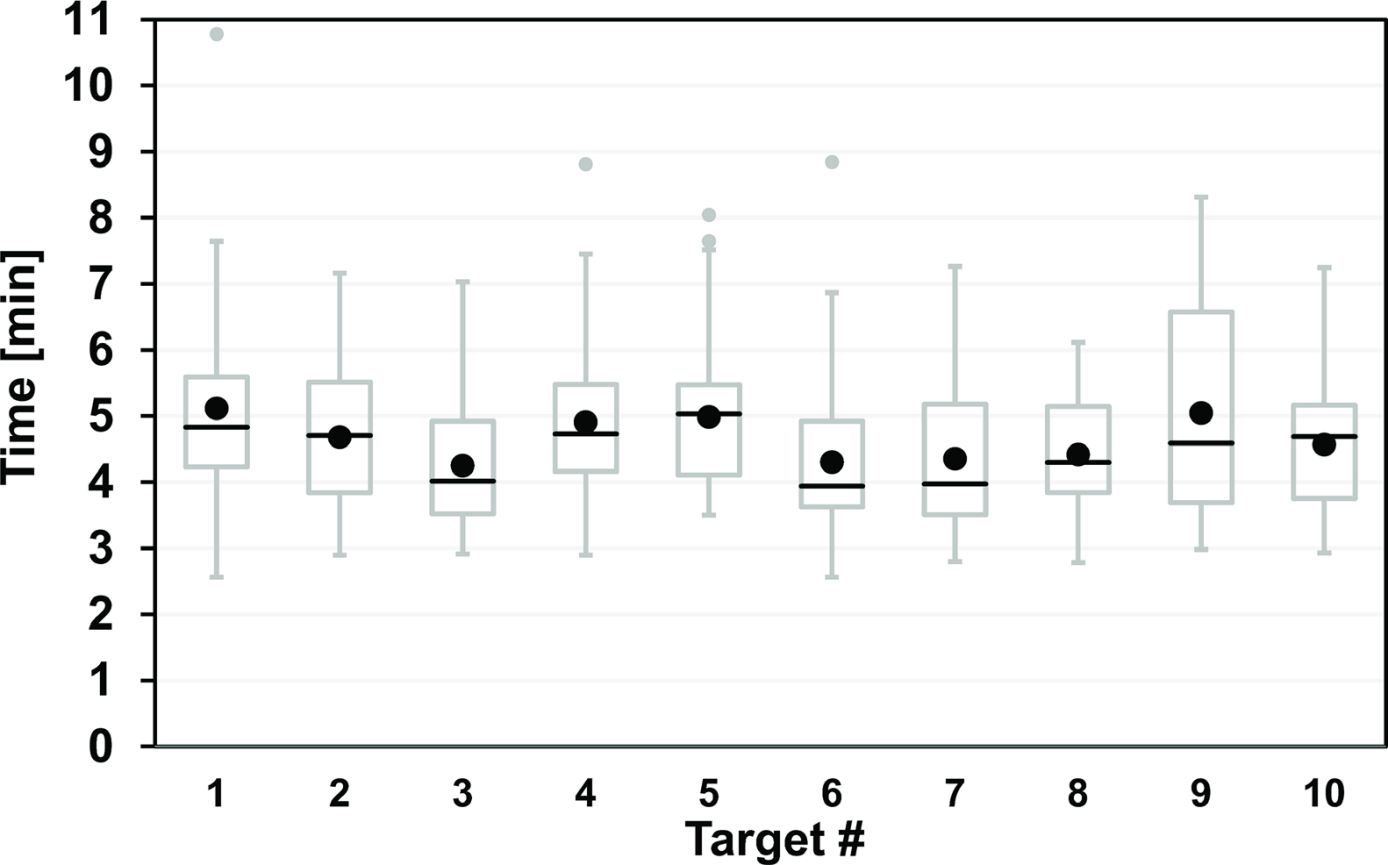
Experimental total biopsy times for 24 operators as a function of target number with variable target depth and ease of access. Each operator approached the targets #1 - #10 in the same order (see Fig 2 for details). Each box with whiskers represents the summary of 24 approaches of a single target by all operators (see Fig 5 for plot details). Four targets had 1–2 upper outliers (gray circles) with times greater than Q3 + 1.5 × IQR. The statistical differences in the biopsy time per target (black circles indicate mean) were not significant (p = 0.065).

The plot of the total biopsy times over all 24 operators against target number is shown in Fig 7. The corresponding mean values range between 4:15 and 5:07 (Δt_max_ = 0:52) and this chronological series features two prominent (differential) increases for targets #4 and #9 (+77% and +73% of Δt_max_). The repeated-measure ANOVA of these biopsy time differences revealed a p-value of 0.065.

### Item Scores

There was no significant between-group difference of the mean item scores (p = 0.093). The rounded Likert scores (*LS*) over all items were 4.37 (AR), 4.19 (RR), 4.16 (MS), and 4.24 (all, Table 1). The majority (9/13) of mean item scores averaged over all groups showed normal to strong agreement (4.13–4.79), in particular, whether the operators would use the system again (*LS* = 4.75, Q12), felt that the outcome justifies the extra effort (4.42, Q10), and trusted the system (4.21, Q13). While average scores were slightly lower with respect to whether the system was self-explaining (3.88, Q04) and regarding the handling of the guiding device (3.67, lowest score of 3.13 by AR, Q06), operators were indifferent about the system's stability against external perturbations (2.79, lowest score of 2.25 by MS, Q07).

## Discussion

The original presentation of the navigation system included a brief assessment of its technical accuracy and a clinical case [15]. The goal of the present work was a clear-cut analysis of the overall diagnostic accuracy, usability and workflow of such an assistance device on a relevant number of experimental biopsies by various medical experts and trainees. Equipment and devices were the same as those used clinically and the range of experimental target depths (17 to 76 mm) was chosen to include some typical insertion lengths for clinical application, for example, the reported 70 mm for liver interventions [11,19]. A key factor for the validity of such a user-dependent assessment was the carefully controlled, standardized fabrication of the phantom.

In the clinical case, correct positioning of the biopsy device is usually evaluated by the MR-visible needle artifact [2,3,10–12,14,19,20]. Its position, size and shape, however, depends on the type of pulse sequence, magnetic field strength and needle orientation with respect to the main field, which is generally seen as a limitation [3,5,8,10,20,21]. A potential offset between MR artifact and true needle position as well as artifact diameters of a few millimeters can be tolerated for the majority of lesions which tend to be larger than that. In a previous work on MR-guided biopsies of 50 liver lesions, however, an average diameter of 15 mm was already considered to be relatively small [3]. Our experimental targets, in comparison, were even smaller (8.5 mm) coming close to the needle artifact size observed here (4–6 mm) and making the usual assessment of positioning accuracy rather difficult. We have therefore decided to judge diagnostic success by the simple presence of green pea material in the otherwise red biopsy sample. This yields an upper limit of the 2D positional accuracy of approximately 4 mm (half the pea diameter) instead of an exact figure. A previous technical assessment has found the average 2D target accuracy to be 2.2 mm and 3.9 mm depending on how far away the reference markers were located from the isocenter [15].

Diagnostic success rates were generally high for all operator groups. The apparent increase seen from medical students via resident to attending radiologists would be in line with the simple picture of better performance by more experienced operators. Statistically, however, there was no significant difference between these groups.

Procedure time is often regarded as a critical factor for MR-guided biopsies [3,11,19,22] and was therefore studied in more detail. The mean times for complete biopsy cycles differed significantly between groups with longer times for less experienced, as expected [7], and the largest between-group differences for the navigation step. The absolute time differences for the puncture step (<3.5 s) were considered negligible but reached significance. The average biopsy cycle times for all professionals (AR and RR) and all operators are considered to be acceptable (4:28 and 4:41 min) with a minimum of only 2:34 min for target #8.

The needle-placement step took around 1.5 min here, which is on the order of those reported for other experimental settings. Meyer et al. [6], for example, have determined puncture times and target errors using a prototype navigation system and real-time MRI in a short, wide-bore 1.5-T system. In a phantom, they achieved a similar mean targeting error (4.0 mm) in about half the time (0:37 min) for experienced operators and 20 trials. In five pigs, the error doubled and the average placement time increased to 5:14 min. Despite low statistical power, these quantitative figures for both settings serve as rough estimators of the loss in accuracy and time introduced by the in vivo setting. Despite some fundamental differences in these approaches (virtual versus real-time images, outside versus inside the bore), a few extra minutes and millimeters are likely to apply for our in-vivo application as well.

A phantom study by Hata et al. [23] has shown that the use of a robotic manipulator significantly reduced the time for needle alignment (from 2:49 to 0:36 min, accuracy 3.0 mm), again with figures being on a similar order of magnitude. Much faster needle placements were experimentally achieved with an augmented reality system outside the bore of a 1.5-T system ranging between 4.2 s (n = 70) in a phantom to 30 s in three pigs (n = 10) [24]. Similarly, the mean needle-to-target distance increased from 2.6 mm to about 10 mm for the animal work. The extent to which high-tech solutions will be developed and clinically deployed still remains to be seen.

The dependence of the average biopsy time with target number or course of time shows no clear trend. The longest biopsy time was observed for the first target although it was the easiest one (short and direct access, not obstructed by vessels). This could be explained by a simple learning effect [11,12,20] which would be in line with the corresponding subjective evaluation (Q05). The two prominent (differential) increases at targets #4 and #9 might be attributed to the increased complexity of the corresponding target approaches (location under vessels) although the overall time differences were not statistically significant (p = 0.065).

The good performance of the navigation system is also supported by the operators’ subjective evaluation. Predominantly high user scores suggest good usability and acceptance of the technique. Regardless of the level of experience, operators considered the solution to be easily understandable and provide additional safety and would also use it again. Replies by medical students to practical items like procedure duration or extra efforts, however, should be interpreted with some caution because of a generally limited experience in that area.

While the average operator rated the system to be marginally self-explaining (3.88, Q04), most users easily understood the working principle (4.79, Q01). We believe that the video instruction has largely contributed to this user rating, in particular because early attempts with simple text instructions were found to be insufficient. A slight agreement was reported for the handling of the articulating arm (3.67, Q06). A later inspection of one of the originally used arms revealed a light corrosion in the fixation mechanism that was probably the result of an early test inside a water bath. In addition, the manufacturer has commented that these elements are now made of stainless steel.

A minimal disagreement was observed with respect to the system's susceptibility against external perturbations (2.79, Q07). Stereotactic guidance systems with reference elements for image registration are generally susceptible to such errors. Displacements of the target region or the patient’s body are inherent limitations of any system and may be addressed by additional patient markers or measures of immobilization [23,24]. The rigidity of the reference, however, depends on the specific organ region and type of procedure. In our case, the flexibility of a reference element for practically the whole body was obtained at the cost of stability. One MRI-specific limitation is the geometric image distortion at the edges of the FOV (closer to the wall of the bore). That issue has already been extensively discussed in the literature and specific figures can be found in [17]. On the other hand, any stereotactic error will be seen in the control images and can be immediately corrected for by a simple update of the navigational road map. A clear advantage of such a floating reference is that navigation is neither affected by repositioning of the 3D digitizer nor by movements of the MR table.

## Conclusions

This study demonstrates high targeting accuracy, usability, and workflow of an "in-and-out" navigation solution for closed-bore scanners in an experimental setting. It must be stressed that during our clinical application, biopsies will never be taken without inspection of the control images. Therefore, the diagnostic accuracies observed here should be regarded as conservative estimates while the total biopsy times are rather lower limits. We believe that this approach is a feasible option for dedicated procedures when following some guidelines. While the clinical performance can only be assessed on real patients, this work provides some valuable findings for a large number of procedures and many different operators that may be relevant for other enabling technologies as well.
